# Mechanisms of Transthyretin Inhibition of IAPP Amyloid Formation

**DOI:** 10.3390/biom11030411

**Published:** 2021-03-10

**Authors:** Sanduni Wasana Jayaweera, Solmaz Surano, Nina Pettersson, Elvira Oskarsson, Lovisa Lettius, Anna L. Gharibyan, Intissar Anan, Anders Olofsson

**Affiliations:** 1 Department of Medical Biochemistry and Biophysics, Umeå University, 901 87 Umeå, Sweden; sanduni.jayakodi@umu.se (S.W.J.); solmaz.surano@gmail.com (S.S.); ninalinneap@gmail.com (N.P.); elos0021@student.umu.se (E.O.); l.eugenia@hotmail.se (L.L.); anna.gharibyan@umu.se (A.L.G.); 2 Wallenberg Centre for Molecular Medicine, Umeå University, 901 87 Umeå, Sweden; intissar.anan@umu.se

**Keywords:** islet amyloid polypeptide, IAPP, amylin, amyloid, transthyretin, TTR, thioflavin T, diabetes

## Abstract

Amyloid-formation by the islet amyloid polypeptide (IAPP), produced by the β-cells in the human pancreas, has been associated with the development of type II diabetes mellitus (T2DM). The human plasma-protein transthyretin (TTR), a well-known amyloid-inhibiting protein, is interestingly also expressed within the IAPP producing β-cells. In the present study, we have characterized the ability of TTR to interfere with IAPP amyloid-formation, both in terms of its intrinsic stability as well as with regard to the effect of TTR-stabilizing drugs. The results show that TTR can prolong the lag-phase as well as impair elongation in the course of IAPP-amyloid formation. We also show that the interfering ability correlates inversely with the thermodynamic stability of TTR, while no such correlation was observed as a function of kinetic stability. Furthermore, we demonstrate that the ability of TTR to interfere is maintained also at the low pH environment within the IAPP-containing granules of the pancreatic β-cells. However, at both neutral and low pH, the addition of TTR-stabilizing drugs partly impaired its efficacy. Taken together, these results expose mechanisms of TTR-mediated inhibition of IAPP amyloid-formation and highlights a potential therapeutic target to prevent the onset of T2DM.

## 1. Introduction

**Figure 1:**
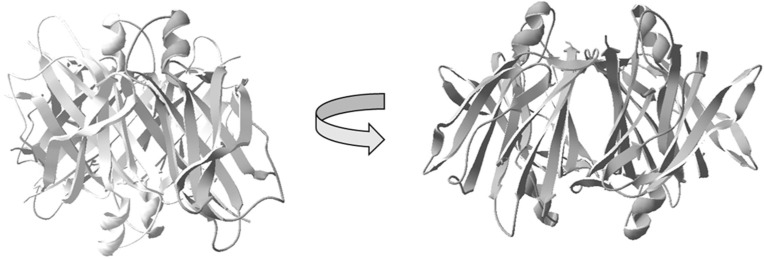
Schematic illustration of the native homotetrameric structure of transthyretin (TTR) [44]. The image illustrates the four subunits assembled to form a hydrophobic central channel amenable for binding of the thyroxine hormone T4, as well as analogs.

Amyloids are characterized by extended β-sheet rich fibrillar structures and are today linked to more than 30 different human disorders, including Alzheimer’s disease, Parkinson’s disease, and type 2 diabetes mellitus (T2DM) [1]. The amyloid component of T2DM is the 37-residue islet amyloid polypeptide (IAPP), also known as amylin, which aggregates and accumulates in the pancreas [2,3]. IAPP is a neuroendocrine hormone, which is produced by pancreatic β-cells and co-secreted with insulin [4]. During normal physiological conditions, IAPP plays an important role in glucose homeostasis and metabolism, regulating gastric emptying and satiety [5,6]. However, under certain pathological circumstances during T2DM development, IAPP aggregates into an amyloid form and deposits in the pancreas. This process is accompanied by a massive loss of β-cells, which is believed to be a consequence of an acute cytotoxic effect induced by amyloid forms of IAPP [7,8]. In the late stages of T2DM, amyloid deposition of IAPP is found in approximately 90% of the patients [9]. The link between IAPP-amyloid deposits and T2DM is further supported by transgenic rodent models engineered to express human IAPP that develop a similar pathology as compared to humans [10,11,12]. The expression of IAPP and insulin are controlled by common promoter elements [13], and both polypeptides are maintained at a ratio around 1:100 (IAPP:insulin). The concentration of IAPP within the secretory granules has been reported to correspond to 1–4 mM [14], which regarding its aggregation propensity is very high considering that IAPP in vitro readily aggregates into amyloid fibrils within the lower μM range [15]. In vivo, the formation of amyloid structures is, however, counteracted by inhibitory and degrading mechanisms, and the pathological accumulation of amyloid can, in essence, be described as an imbalance between the rate of amyloid formation and the rate of its degradation. Within this context, it has been shown that both the low pH in the granules [16,17], pro-IAPP as well as the crystalline form of insulin, counteract the amyloid-formation of IAPP [18]. Regarding amyloid in general, a small set of proteins have also been shown to interfere with the rate of amyloid formation, and although most studies have been performed on the Aβ peptide linked to Alzheimer’s disease, these amyloid-interfering proteins frequently have a low target-specificity and may therefore act on several different amyloids. A few examples from this group of amyloid-interfering proteins includes the BRICHOS domain [19,20,21], apolipoprotein J (ApoJ) [22,23], apolipoprotein E (ApoE) [24,25,26,27,28,29], and transthyretin (TTR) [30,31,32,33,34]. The anti-amyloidogenic properties of TTR on Aβ amyloid formation have gained much attention due to its potential protective role in Alzheimer’s disease, and beneficial effects have been shown on both cellular and animal models [32,35,36,37,38,39,40,41]. Concerning amyloid-formation of IAPP, it was recently shown that a designed monomeric form of TTR could interfere with the process [42]. TTR is produced in the liver, the choroid plexus of the brain, retina of the eye, and interestingly, it is also abundantly expressed by the pancreatic β-cells [43]. The expression of TTR within the pancreatic β-cells, suggests that it may have a protective role against T2DM and hence highlights the need for an elucidation of its inhibitory mechanism on IAPP amyloid formation. The native form of TTR is a 55 kDa homotetramer, predominantly constituted by β-strands, (a schematic structure of TTR is illustrated in Figure 1).

In vivo, TTR acts as a transporter-protein of thyroxine T4, where the hormone binds within the central-core of the tetramer [45]. TTR can bind the retinol-binding protein and thereby also acts as an indirect carrier of retinoic acid [46].

Intriguingly, in analogy to both IAPP and Aβ, TTR is also associated with intrinsic amyloidogenic properties, which are linked to familial amyloid polyneuropathy (FAP) and familial amyloid cardiomyopathy (FAC) in humans. The dual properties of TTR as both an amyloidogenic protein and an amyloid inhibitor are not fully elucidated and may appear paradoxical. Anti-amyloidogenic properties of amyloid-forming proteins have, however, previously been reported [47], and the amyloid-interfering effect could possibly even require similar properties between the target and inhibitor.

Concerning the intrinsic amyloidogenic properties of TTR, a dissociation of the tetramer is a rate-limiting step in the process [48,49]. Interestingly, the conversion of native TTR into its amyloid form can be effectively prevented by tetramer-stabilizing drugs, and this reaction can be controlled through the use of small ligands binding at the position of the natural ligand-binding site of the thyroxine T4 hormone [49]. Several stabilizing TTR ligands have been identified and are currently in clinical use to slow-down the progression of FAP [50,51,52,53,54].

In the present study, we have elucidated the properties of TTR that correlate with its ability to interfere with IAPP amyloid formation. We have studied the effect both in terms of the intrinsic TTR-stability, based on a range of TTR variants, as well as the effect of extrinsically added TTR-stabilizing drugs. We have also investigated the interfering effect of TTR within the low pH environment of the IAPP containing granules of the pancreatic β-cells, which notably also varies as a function of blood glucose levels [55]. We show that the efficacy of TTR correlates inversely with its thermodynamic stability while no such correlation is observed regarding the dissociation rate of the TTR-tetramer. Within this study, we also expose that TTR, in contrast to its effect on Aβ, effectively inhibits IAPP fibril elongation. The interfering effect is preserved also at the low pH corresponding to the environment within the pancreatic granules. However, the addition of TTR-stabilizing drugs, at both neutral and low pH partly impaired its effect. The results highlight TTR as an interesting target in the regulation of IAPP amyloidosis and increasing its efficacy potentially could possibly prevent or postpone the onset of T2DM.

## 2. Materials and Methods

### 2.1. TTR, ApoE, and IAPP

Recombinant TTR variants, recombinant Apolipoprotein E (ApoE) ε3 (1-299), and synthetic human IAPP 1-37 were obtained from AlexoTech AB (Umeå, Sweden). The quality of the proteins was verified by LC-MS before analysis. The employed IAPP contained a disulfide bridge between the positions Cys2 and Cys7 and was amidated at its C-terminal.

### 2.2. Size-Exclusion Chromatography

The lyophilized IAPP was dissolved in 5% acetic acid and 150 mM sodium chloride while all TTR variants and ApoE ε3 were dissolved in phosphate-buffered saline PBS. Before use, all polypeptides including IAPP, ApoE ε3, and the different TTR variants were subjected to size-exclusion chromatography (Superdex 75 10/300 GL; GE Healthcare, Chicago, IL, USA) equilibrated with 20 mM phosphate buffer containing 150 mM NaCl (PBS).

### 2.3. Thioflavin T Fluorescence Assay

Amyloid formation of IAPP at neutral pH was performed at 5 μM peptide concentration in PBS supplemented with 40 μM Thioflavin T (ThT) (Sigma-Aldrich, Saint Louis, MO, USA). Amyloid formation monitored at pH 4.5 was performed at 10 μM in 25 mM citric acid buffer containing 150 mM NaCl. All fluorescence measurements were performed at 37 °C in 384 microtiter-plate, (black walls and clear bottoms, Nunc) using a FLUOstar Omega microplate reader (BMG Labtech GmbH, Ortenberg, Germany) with an excitation wavelength of 430 nm and an emission wavelength of 480 nm, the samples were shaken for 1 s at 100 rpm every 30 min before reading. All experiments were performed in triplicate or more and each experiment has been verified three times or more. For experiments using diclofenac and luteolin, the drugs were dissolved in DMSO. The working solution of the drugs, as well as the control, contained 1% DMSO.

### 2.4. Probing IAPP-Amyloid Elongation

Monomeric IAPP at a concentration corresponding to 10 μM in PBS supplemented with 40 μM ThT was prepared 100 μL/well in 96 microtiter-plate (black walls and clear bottoms, Corning, New York, NY, USA, Cat. No. 3881). At the indicated time points, the reader was paused and respective concentrations of TTR L12P, as well as ApoE ε3, purified using size-exclusion chromatography, were added. TTR L12P and ApoE ε3 were added from a 20× concentration to minimize dilution. From the respective stock, 5 μL of the respective peptide was added to a well containing IAPP (100 μL). The control wells (no TTR) contain 5 μL of PBS [55].

## 3. Results

The conversion of monomeric IAPP into its amyloid fold has been extensively studied and follows a nucleation-dependent mechanism [56]. A nucleation-dependent mechanism can in general be described by a sigmoid curve initiated by a lag-phase, where the formation of fibrils is still low and where the spontaneous formation of oligomeric nuclei controls the rate of the reaction. As the reaction proceeds, the lag-phase is converted into a logarithmic phase, where elongation through the incorporation of monomers now dominates. The template-dependent incorporation of monomers into the fibril end has a significantly lower energy-barrier than the nucleation and is consequently much faster [57]. New ends for the incorporation of monomers can form as a function of fibril-breakage but also as a result of fibril-catalyzed secondary nucleation where the surface of already formed fibrils may catalyze the formation of new nuclei [58]. As the number of monomers available for incorporation becomes reduced, the reaction approaches a steady-state and equilibrium between mature fibrils and free monomers. In a recent publication, the specific microscopic events of IAPP amyloid formation were studied and found to fit with a model based on primary nucleation + elongation and surface-catalyzed secondary nucleation, but not fibril breakage [59].

**Figure 2:**
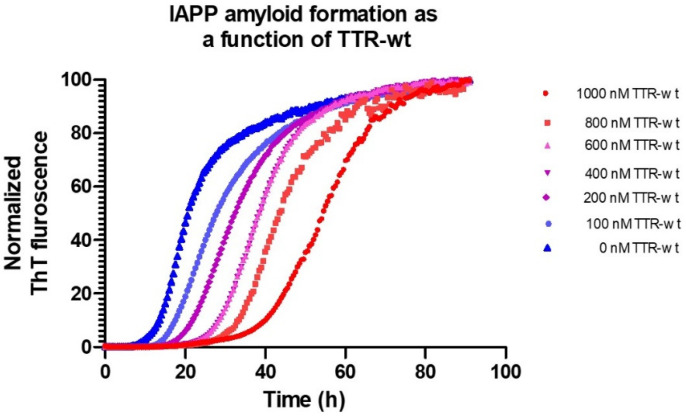
Amyloid formation of 5 μM IAPP at 37 °C, pH 7.5 in phosphate-buffered saline monitored by Thioflavin T as a function of various concentrations of TTR-wt.

Using microtiter-based ThT-assays, the effect of different conditions as well as intervening agents can be studied in detail. Figure 2 shows the process of IAPP fibril-formation, inhibited by the TTR wild-type, (seen as a prolongation of the lag-phase), under physiological pH and ion-strength.

### 3.1. Evaluating the Inhibiting Propensity of TTR as a Function of Its Intrinsic Stability

**Figure 3:**
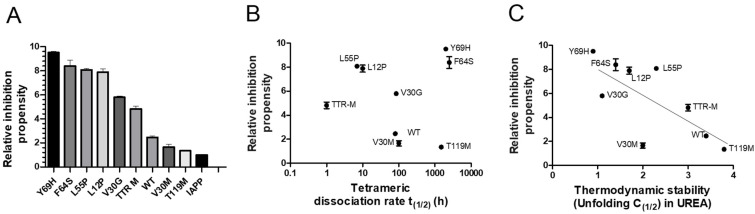
ThT analysis of the relative islet amyloid polypeptide (IAPP)-amyloid inhibiting propensity exerted by TTR-wt, L12P, V30M, V30G, L55P, F64S, Y69H, T119M, and the monomeric variant TTR-M. The lag-phase of the TTR-wt versus control was set to 1.0, and the effect from all other TTR-variants were normalized accordingly (**A**). The relative inhibitory effects of the specific TTR-variants are plotted as a function of kinetic stability represented by the dissociation-rate of the tetramer (**B**) and thermodynamic stability (**C**). An inverse correlation between the inhibitory effect and thermodynamic stability is shown (r^2^ = 0.53 and *p*-value < 0.0001).

The properties required for TTR to effectively interfere with IAPP amyloid-formation are largely unknown. Regarding Aβ amyloid formation, the stability of TTR has, however, been shown to influence its effect. The stability of TTR can be divided into a thermodynamic and kinetic component. The thermodynamic stability is represented by the static equilibrium between its folded and unfolded state. A rate-limiting step regarding the unfolding of TTR is, however, mediated by dissociation of its tetrameric integrity [60]. To elucidate the influence of TTR-stability regarding its effect on IAPP-amyloid formation, a range of TTR variants, differing in thermodynamic and kinetic stabilities were compared. The results illustrated in Figure 3A, show the relative IAPP-amyloid inhibiting propensity of TTR-wt, L12P, V30M, V30G, L55P, F64S, Y69H, T119M, and the monomeric variant TTR-F87M/L110M (TTR-M) [61]. The relative time to reach the midpoint of the logarithmic phase (t_(1/2)_) is measured. For comparison, the t_(1/2)_ of IAPP amyloid-formation reaction in absence of TTR is set to 1.0, and the relative inhibiting propensity of all TTR variants is related to this value. The results show that several TTR variants display a significantly potentiated effect as compared to TTR-wt, thus displaying considerable variation. Both the thermodynamic stabilities and the kinetic dissociation-rates with regards to the employed TTR variants are known from previous studies [61,62], and consequently, the inhibiting propensity can be related to these values. Plotting the relative inhibiting propensities against the kinetic (Figure 3B) and the thermodynamic (Figure 3C) properties of TTR, exposes an inverse correlation between thermodynamic stability and the interfering ability of TTR upon IAPP amyloid formation, while no such correlation could be observed regarding its kinetic stability.

### 3.2. Probing the Ability of TTR to Interfere with IAPP Fibril Elongation

**Figure 4:**
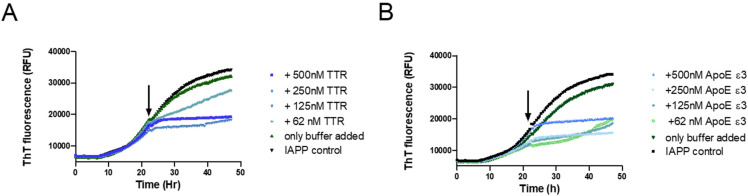
Probing the ability of TTR to inhibit the elongation process during IAPP amyloid formation. A total of 5 μM of IAPP was incubated in phosphate-buffered saline and monitored for amyloid formation with the ThT fluorescence assay. At the timepoint indicated by the arrow, the respective proteins were added at the indicated concentrations: TTR L12P (**A**) recombinant Apolipoprotein E (ApoE) ε3, serving as a positive control (**B**).

To further elucidate the mechanistic path of IAPP-amyloid inhibition by TTR, its effect on IAPP elongation was selectively probed. This can be accomplished by targeting the logarithmic phase of amyloid formation where the contribution from nucleation can be neglected in favor of elongation [57,63]. By the addition of the inhibitory agent within the logarithmic phase, and subsequently monitoring the curvature, it can be determined whether elongation is affected or not. This approach has previously been used in two independent studies showing that Apolipoprotein E (ApoE) efficiently inhibits both the elongation of Aβ- [64] and IAPP-amyloid [15]. We have previously also used this approach to show that TTR is unable to inhibit the elongation of Aβ amyloid [34]; a result that was independently verified recently [65]. In order to accomplish a clear result also at a high substoichiometric ratio, the potent TTR variant L12P was employed. The setup was probed with ApoE as a positive control to validate the assay. The result displayed in Figure 4 shows that TTR, in contrast to its effect on Aβ, efficiently inhibits fibril elongation of IAPP.

### 3.3. Probing the Inhibiting Effect of TTR at Neutral and Low pH, and the Effect of TTR-Stabilizing Drugs

**Figure 5:**
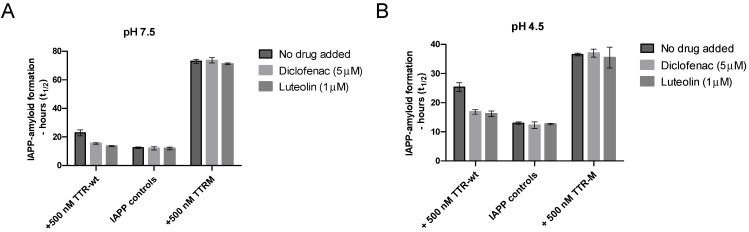
Inhibition of IAPP amyloid formation by TTR as a function of TTR-stabilizing ligands at neutral and low pH. Amyloid formation of IAPP incubated in PBS (pH 7.5) or 25 mM citric acid pH 4.5, 150 mM NaCl, was probed against 500 nM of TTR-wt as well as TTR-M in the presence versus absence of the tetramer stabilizing drugs luteolin (1 μM) and diclofenac (5 μM). Figure (**A**) illustrates the result at neutral pH at 5 μM of IAPP. Figure (**B**) illustrates the results at pH 4.5 (note that 10 μM of IAPP was used in the setup at low pH). A significant reduction of the inhibiting propensity of the TTR stabilizing drugs was observed regarding the tetrameric TTR-wt, *p*-value < 0.005 (unpaired *t*-test), both at neutral and low pH. TTR-M, which is unable to bind to the ligands, was unaffected at neutral and low pH.

The environment within the IAPP-secretory granules also differs from the extracellular environment regarding pH, which in vivo is approximately 5 [55]. A low pH has been found to partly suppress IAPP amyloid-formation [66], and similarly, TTR is also well-known to be sensitive to changes in pH [48]. Regarding TTR, the structural changes induced by low pH can, however, be partly prevented by TTR-tetramer stabilizing drugs [49]. To investigate whether the ability of TTR to prevent amyloid formation is maintained at low pH and whether the addition of TTR-stabilizing drugs can modulate the effect, we compared the ability of TTR-wt to interfere with IAPP amyloid formation in the absence and presence of the two stabilizing agents diclofenac [67] and luteolin [68], at both neutral pH and pH 4.5, see Figure 5. The monomeric variant TTR-M, unable to bind the stabilizing ligands, was included as a control.

The results show that the inhibiting effect of TTR on IAPP-amyloid formation can also be observed at a low pH but that the addition of TTR-stabilizing ligands partly impairs the effect at both neutral and low pH. Regarding the monomeric variant TTR-M, which is unable to bind the ligands diclofenac and luteolin, no effect was observed as a function of adding the drugs. Due to a comparatively low ThT fluorescence upon binding of IAPP fibrils at low pH, the concentration of the peptide was raised to 10 μM regarding all analysis performed at low pH, to maintain the desired accuracy of the measurements. This results in an overall shortening of the lag-phase. A low pH has previously been found to partly impair amyloid formation of IAPP [66] and to emphasize that this is indeed also observed within our experimental setup, a figure has been added to Supplementary materials Figure S1.

## 4. Discussion

In analogy to ApoE that can interfere with the amyloid formations from several different amyloidogenic proteins [15,69,70], TTR also appears to have generic amyloid-interfering properties and has recently been shown to interfere with the amyloid-formation of the bacterial proteins CsgA [71] and HypF-N [32]. Regarding Aβ, the mechanistic effects of TTR have been extensively studied and it is now well established that it selectively targets the oligomeric assemblies of Aβ [34,42,65,72,73], while it does not interfere effectively with Aβ-fibril elongation [34,65]. Regarding the inhibition of Aβ-amyloid formation by TTR [31], it can be concluded that the highly stable T119M, as well as K15A, are less efficient than the less stable variants V122I, V30M, and TTR wild-type. The inhibiting effect on Aβ was previously also shown to be partly impaired by the addition of the TTR stabilizers [31].

### 4.1. The Inhibition of IAPP Amyloid-Formation by TTR Correlates with Its Thermodynamic but Not Kinetic Stability

Within the present work, we show that TTR variants with a kinetically stable tetrameric form also can interfere with IAPP, similar to the recently described engineered monomeric TTR-M [42]. The influence of TTR stability is, however, obvious, and TTR-M is a more potent inhibitor than TTR wt. This raises the questions of whether tetrameric dissociation and/or the exposure of normally buried sequences are required for the inhibiting activity.

Today, more than 140 mutations in TTR have been identified in vivo [74] and biophysical analysis of a selection of these TTR variants exposes a significant variation regarding their stability. A static equilibrium between its folded and unfolded state, measured through urea-denaturation studies, here defines its thermodynamic stability. Under denaturing conditions, the thermodynamic unfolding of TTR is kinetically limited by a dissociation of the tetramer and, the unfolding kinetics as a consequence correlates with the dissociation of its tetrameric integrity [49]. Dissociation under non-denaturing conditions is, however, not associated with a complete unfolding and, e.g., the monomeric TTR-M retains a similar structure compared to the native fold within the tetramer. It notably also requires further denaturation to aggregate into TTR-amyloid [61].

Within the present study, a range of TTR-mutants differing in both their thermodynamic and kinetic stability was investigated regarding their ability to interfere with IAPP-amyloid formation. Using the available biophysical data [61,62] we show that the ability of TTR to interfere with IAPP amyloid formation displays an inverse correlation to its thermodynamic stability, while no such correlation can be seen with regards to its kinetic stability. Notably, the thermodynamically unstable but kinetically highly stable variants Y69H and F64S, both display a very strong amyloid-interfering effect on IAPP. While T119M having similar kinetic stability but with much higher thermodynamic stability is essentially inert. Since the monomeric concentration of both Y69H, as well as F64S, is very low under native conditions this strongly suggests a partial exposure of the TTR polypeptide that normally is buried within its native fold. The occurrence of such partial unfolding is supported by previous studies using nuclear magnetic resonance (NMR) where a different surface exposure between different tetrameric variants of TTR has been shown using both Hydrogen-Deuterium (H/D) exchange [75] and relaxation dispersion experiments [76,77]. The exposure of normally hidden parts of TTR has also been shown using the conformational specific antibody MAB_(39–44)_, which normally only binds to unfolded TTR or its amyloid fold but where a strong reactivity is obtained to the tetrameric form of Y78F [78]. As a consequence, we propose a hypothesis where a partial unfolding event of TTR, independent of TTR-tetramer dissociation, mediates the inhibitory effect. The specific areas for this interaction will require further studies and we also cannot at this point exclude that the interaction and complex formation between TTR and the IAPP-assemblies facilitates a subsequent more pronounced unfolding and possibly also dissociation of the tetramer.

### 4.2. TTR Prevents Elongation of IAPP Fibrils

An amyloid-interfering agent can mediate its effect at different positions in the reaction path of amyloid formation. These are represented by (i) binding and sequestering of the monomer; (ii) binding and sequestering of nuclei; (iii) inhibition of secondary nucleation via blocking of catalytic sites; (iv) blocking of elongation; and (v) inhibition of fibril breakage. In a recent paper, it was shown that the formation of IAPP amyloid fits with a model involving primary nucleation + elongation and surface-catalyzed secondary nucleation, but not fibril breakage [59]. We can here show that TTR interferes with the process of elongation. The experimental approach is based on the rationale that elongation strongly dominates over both primary and secondary nucleation during the logarithmic phase [79,80]. The addition of an inhibitor during the logarithmic phase as a consequence predominantly probes the process of elongation. To investigate if TTR could interfere with IAPP elongation the potent L12P variant was used. The results show an efficient abortion of the logarithmic phase upon the addition of TTR and an effect that is notable also at a 1:80 (TTR:IAPP) ratio. The result implicates that the inhibitory effect is mediated via binding of TTR to the fibrils and shows that the inhibition is not mediated by a sequestering of the IAPP monomer. Using this approach, it was recently shown that ApoE effectively inhibits the elongation of both Aβ [64] and IAPP [15]. ApoE was therefore included as a positive control to validate the system. The finding that TTR interferes with IAPP fibril elongation notably exposes a discrepancy between IAPP and Aβ since TTR is unable to inhibit the elongation of Aβ, as recently shown in two independent studies [34,65].

As illustrated in Figure 2; Figure 3, the presence of TTR from the start of the reaction results in a concentration-dependent prolongation of the lag-phase. In addition, here the effect can be observed at highly substoichiometric ratios of TTR strongly supporting that assemblies of IAPP represent the primary target rather than a sequestering of the monomer. The shift in lag-phase fits with a model where either primary nucleation or fibril elongation is impaired [81]. Although a likely explanation is that both of these are affected, an elucidation of the specific mechanism requires more specialized experiments and remains to be elucidated by future investigations.

### 4.3. TTR Prevents IAPP Amyloid Formation at Low pH but the Effect Is Impaired by Stabilizing Ligands

In vivo, IAPP is stored within the secretory granules of the pancreatic β-cells at a concentration around 1–4 mM [14], which compared to its extracellular concentration of <1 nM [82] renders the interior of the granules a likely site for the formation of IAPP-amyloid.

The pH within the IAPP-secretory granules is acidic and is frequently around pH 5. Notably, both TTR and IAPP are known to be strongly affected by perturbations in pH. Regarding IAPP, lowering the pH is correlated with a lower propensity to aggregate [83]. This result is also corroborated by us and is presented in Supplementary files Figure S1. TTR, in contrast, results in an increased aggregation rate as a function of low pH [48,84]. Given that IAPP amyloid formation likely is an intracellular event (occurring within the IAPP containing granules of the β-cells), it is of interest to investigate the inhibitory effect of TTR also at a low pH. We have here evaluated the effect at pH 4.5, which represents a pH known to induce significant alteration of TTR [48]. At pH 4.5, the major pH-mediated alterations in the rate of IAPP have occurred [83]. It is also close to the physiological pH range found in vivo which is around 5 but which notably also varies as a function of blood-glucose [55]. The results show that TTR can impair the effect of IAPP-amyloid formation also at low pH. A correspondingly stronger effect, as observed at neutral pH, can also be seen for TTR-M, supporting the notion that exposure of epitopes buried within the native structure is required for an efficient inhibition.

Ligand stabilization of TTR, using the site for thyroxin-T4, is today an established therapeutic approach to treat FAP [85]. The binding of a ligand to TTR has, however, previously been shown to partly impair its anti-amyloidogenic properties regarding the inhibition of Aβ amyloid formation [31]. We here show that this effect is also pronounced on IAPP. In this study, two different TTR-ligands represented by luteolin [68] and diclofenac were evaluated. It should be clarified that TTR displays negative cooperativity upon ligand binding and only the first ligand binds effectively. Luteolin represents a strong TTR-binder with a K_D_ around 20 nM, and using 1000 nM concentration of the drug and 500 nM of TTR, we ensure both a stoichiometric excess and a high level of saturation. The K_D_ of diclofenac is around 500 nM [53], and so in order to ensure a high level of saturation, 5 μM of diclofenac was employed. The results show that ligand binding to TTR interferes with IAPP-amyloid formation at both neutral pH and pH 4.5. The TTR-M, which is unable to bind the tetramer-stabilizing ligands, was unaffected at both neutral pH and pH 4.5, which also validates the system.

The poor correlation between dissociation kinetics and the ability to inhibit amyloid-formation of IAPP, shown by the different variants in Figure 3A–C, suggests that the impairing effect of TTR is likely mediated by the ability to expose parts of the polypeptide that normally are buried within the native fold. As stated above, we propose a hypothesis where this alternative structure can be acquired also within the tetramer. The impairing effect from stabilizing ligand is possibly mediated by reducing the ability of the protein to partly unfold.

## 5. Conclusions

In this study, we have characterized the properties of TTR regarding its ability to interfere with IAPP-amyloid formation. We expose an inverse correlation between thermodynamic stability and the ability to interfere with IAPP-amyloid formation while no such correlation could be noted regarding the kinetic stability of the TTR-tetramer. The inhibiting effect is observed also at a highly substoichiometric ratio showing that TTR does not mediate its effect via binding and sequestering of the monomer but rather binds to assemblies of the peptide. This is further supported by the finding that it efficiently impairs fibril elongation at highly substoichiometric ratios which also implicates binding of TTR to the fibrils.

From a mechanistic point of view, we propose that a partial unfolding of TTR is required for its amyloid-interfering effect and that exposure of normally buried parts of the molecule is required. The results suggest that the partial unfolding can be mediated also within the tetramer. It is, however, yet not possible to conclude if a subsequent unfolding occurs upon the interaction between TTR and the IAPP, and if the formed assembly is associated with dissociation of its tetrameric integrity.

**Figure 6:**
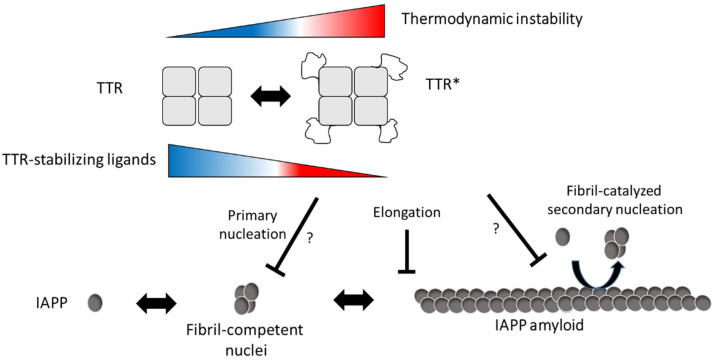
Schematic illustration of the suggested inhibitory mechanism by TTR on IAPP amyloid-formation. In accordance with [1], our results suggest that TTR targets assemblies of IAPP and that its amyloid-interfering effect is not based on the binding and sequestering of IAPP monomers. In addition, we observe an inverse correlation between thermodynamic stability and the ability to interfere with IAPP amyloid formation, while no such correlation was observed regarding the kinetic stability, represented by the dissociation-rate of the tetramer. We further show that TTR, in analogy to ApoE, can impair fibril elongation of IAPP which also implies that it binds to the fibrils. Although targeting of primary nuclei can be anticipated, it cannot be unambiguously distinguished from the effect of fibril elongation which both result in the observed prolongation of the lag-phase. It is from these data also not possible to show if the binding of TTR to the fibrils also impairs fibril-catalyzed secondary nucleation. In conclusion, we propose that the interfering effect of TTR requires exposure of normally buried parts of TTR and indicates an equilibrium between its native fold and a partially unfolded form (TTR*). We propose that exposure of these areas can occur also within the tetrameric state of TTR but cannot exclude that the interaction between IAPP and TTR results in a subsequent unfolding as well as tetramer dissociation. We show that the addition of TTR-stabilizing ligands can partly impair the inhibiting effect and based on the model we propose that ligand binding prevents the unfolding into TTR*.

We also show that the efficacy of TTR can be modulated using TTR-stabilizing ligands and that they impair the effect at both neutral and low pH. A mechanism where binding of the ligands impairs a partial unfolding of the tetramer can be anticipated but needs to be further investigated. A model describing our hypothesis by which mechanisms TTR interferes with IAPP amyloid formation is proposed in Figure 6.

Given the specific expression of TTR within the pancreatic β-cells TTR and its amyloid-interfering effect, a physiological role can be anticipated. TTR hence provides an interesting target to modulate IAPP amyloid-formation and thus also a potential route to prevent or postpone the onset of T2DM. We here present the overall properties required for TTR to inhibit IAPP amyloid-formation and interestingly also that its effect can be partly impaired by ligands. The environment within the secretory granules is nevertheless complex and an interesting prospect for the future is to follow up these findings in an in vivo model to fully elucidate the role of TTR and its therapeutic potential.

## Data Availability

Not applicable.

## Supplementary Materials

The following are available online at https://www.mdpi.com/2218-273X/11/3/411/s1, Figure S1: Amyloid formation of IAPP as a function of pH illustrated by the midpoint of the ThT curve after incubation of 10 μM IAPP in 150 mM NaCl supplemented with 40 μM ThT (Sigma-Aldrich, Saint Louis, MO, USA) containing 25 mM phosphate buffer (pH 7.0, 6.5 and 6.0) and 25 mM citric acid, (pH 5.5 and 5.0) respectively.
