# Supplementary material for: Mechanisms of Transthyretin Inhibition of IAPP Amyloid Formation
Source: Biomolecules. 2021 Mar 10;11(3):411. doi: 10.3390/biom11030411 (PMC8001701; doi:10.3390/biom11030411)
Supplement: Supplementary file 1 [file biomolecules-11-00411-s001.pdf]

Supplementary material

Figure S1

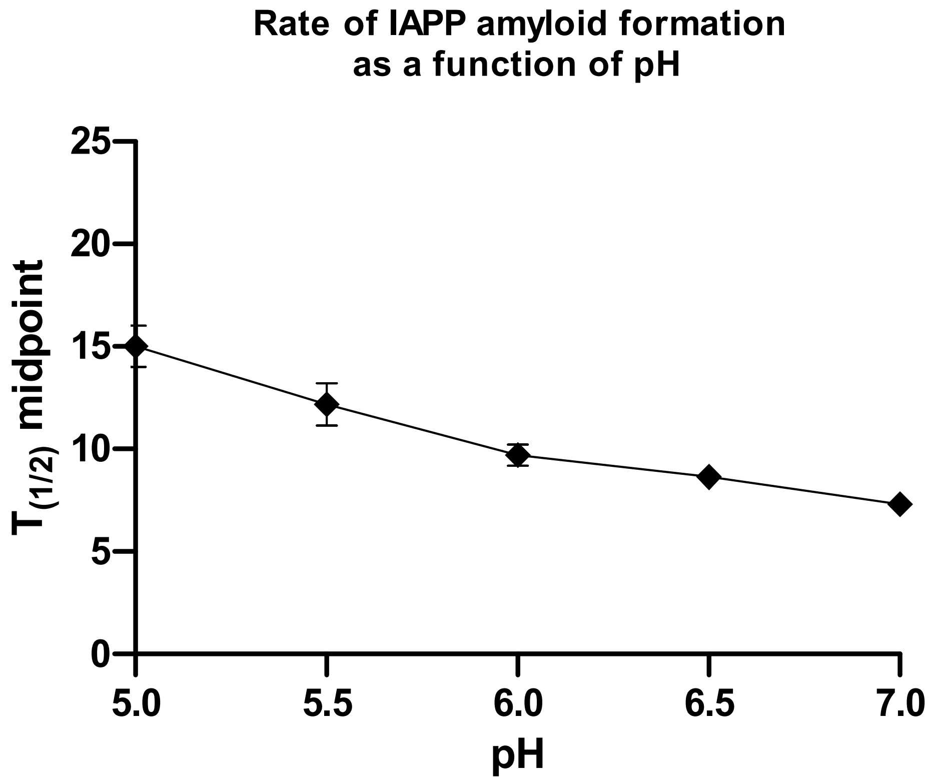

Figure S1. Amyloid formation of IAPP as a function of pH illustrated by the midpoint of the ThT curve after incubation of 10µM IAPP in 150 mM NaCl supplemented with 40 µM ThT (Sigma-Aldrich, Saint Louis, MO, USA) containing 25 mM phosphate buffer (pH 7.0, 6.5 and 6.0) and 25 mM citric acid, (pH 5.5 and 5.0) respectively. All fluorescence measurements were performed at 37°C in 384 microtiter-plate, (black walls and clear bottoms, Nunc) using a FLUOstar Omega microplate reader (BMG Labtech GmbH, Ortenberg, Germany) with an excitation wavelength of 430 nm and an emission wavelength of 480 nm, the samples were shaken for 1 second at 100 rpm every 30 minutes before reading.
